# The Complex Interplay Relationship between HCV Infection, Direct-Acting Antiviral Therapy, and Hepatocellular Carcinoma Occurrence

**DOI:** 10.3390/cancers15215196

**Published:** 2023-10-28

**Authors:** Luisa Cavalletto, Erica Villa, Liliana Chemello

**Affiliations:** 1 Department of Medicine-DIMED, University-Hospital of Padova, 35128 Padova, Italy; luisa.cavalletto@unipd.it; 2 CHIMOMO Department, University of Modena and Reggio Emilia, 41121 Modena, Italy

The new direct-acting antivirals (DAAs) for chronic hepatitis C (CHC) are highly effective, despite the short duration of treatment, and very tolerable. Many studies have demonstrated the benefits achieved in the majority of patients treated with DAAs (2015), due to the significant reduction in liver morbidity and mortality observed [1,2], but the question arises as to whether this favorable effect occurs *for everyone*, and *for how long*?

As part of this editorial, we have selected three compelling articles recently published in the journal *Cancers* [3,4,5], which address these unsolved questions. The selected articles focus on the long-term outcomes of individuals who have recovered from HCV infection, particularly focusing on those subjects who, despite achieving a long-term response (LTR), still remain at risk of progressive liver disease with the potential development of hepatic decompensation [6] or hepatocellular carcinoma (HCC) [7].

The first paper by Flisiak et al. [3] presents a real-world experience from a multicenter Polish database, *the AMBER study* [8], of 192 cases, followed up for 5 years, of whom 95% had the HCV-1 genotype and 57.3% had liver cirrhosis. The annual HCV-related mortality rate at 2 years vs. 5 years significantly increased from 1.5 vs. 3.7 per 100 person/year. Twelve de novo HCC cases were diagnosed (75% of whom had liver cirrhosis). On the other hand, after DAA administration, the majority of the patients returned to normal hepatic function, with 95%, 80% and 88% of cases normalizing their albumin, bilirubin and INR level, respectively, during follow-up (FU). In 78 cases with cirrhosis, liver fibrosis reversed with a significant decrease in liver stiffness (LS) value, measured by vibration-controlled transient elastography (TE), from 17.9 kPa at baseline to 9.65 at 5-year FU. This study, like many others published, not only highlights the benefit of HCV treatment but also the persisting risk of HCC following viral elimination.

The PITER study, based on an Italian multicenter prospective data collection [9], showed similar results, with an overall HCC incident risk of 2.45/100 p/y at 36 months. The highest risk was associated with Child class B (log-rank test *p* < 0.01 through KM analysis).

The importance of Child class B was further confirmed in a study on 139 HCV-related cirrhotic patients, whose condition was independent of a previous history of HCC [10]. The same study showed the relevance of a decrease lower than 30% in LS value after DAA administration as a risk factor for HCC development. This finding further emphasizes the importance of measuring LS by TE to define the stage of disease, as already highlighted in many clinical practice guidelines [11]. Both studies, however, also draw attention to the fact that obtaining an LTR is not entirely protective against HCC development [3,10]. There remains a “*sword of Damocles*” on the head of cases with LTR, particularly in those with F3–F4 staging according to the Metavir fibrosis classification.

Therefore, in response to this issue, Caviglia et al. [4] attempted to identify the best predictors of HCC onset in 575 patients with liver cirrhosis and LTR after DAA administration with an FU of 44.9 (27.8–58.6) months. They assessed the diagnostic accuracy of five currently used non-invasive scoring systems (FORNS, APRI, FIB-4, ALBI, and aMAP). In this study, 57 of the 575 (9.9%) subjects developed HCC, at a median time to tumor onset of 29.3 (16.4–41.1) months after HCV eradication. Of the patients, 65% were at the earlier class 0-A according to the Barcelona Clinic Liver Cancer (BCLC) staging system, while showing features of a severe liver disease (i.e., low platelet count and albumin level and high bilirubin value), and Child class B and esophageal varices were recorded in 16% and 50% of cases, respectively. The ALBI score was found to have the best predictive performance by the C-index (0.70, 95% CI 0.62–0.77) 12 weeks after the end of therapy and was the only score independently associated with de novo HCC development during multivariate analysis.

Despite the fact that LTR after DAA administration has been found to be associated with slowing down the progression of liver disease in many cases with advanced fibrosis, those with clinically significant portal hypertension have probably passed *a point of no return*, which leads to an unfavorable long-term disease outcome in the majority of cases. The early identification of these cases may be predictable through the non-invasive TE measurement of spleen stiffness (SS) or through the application of combined scores, such as the LS–spleen diameter to platelet score (LSPS) [12,13]. These tools may ameliorate the application of cost-effective strategies for continuous HCC surveillance in cases with LTR after DAA administration. Other strategies to identify at-risk patients could also lie in the utilization of scoring systems that put together independent HCC risk predictors through use of a nomogram, or better, through the use of machine learning, to model patient-tailored HCC risk [9,14].

Finally, the retrospective, multicenter collection by Yeh et al. [5] offers us the opportunity *to face the other side of the coin* and conclude our perspective on this topic. The authors present a retrospective-multicenter analysis of 1389 cases with HCC, which includes patients who developed HCC who were either viremic (78%) or non-viremic following DAA therapy (301 cases) to compare the characteristics and survival curves between the cases. The non-viremic status in the cases with HCC occurrence correlated significantly with higher survival (153.3 vs. 55.6 months) in comparison with the HCC viremic cases, with them also showing a lower rate of obesity, metabolic dysfunction-associated steatotic liver disease (MASLD), and liver cirrhosis and better liver function and an earlier stage of HCC (BCLC stage 0-A in 74.8%). This fact certainly also determines the chance of receiving curative tumor treatment compared to viremic cases with HCC (HR 0.60, 95% CI 0.49–0.73). By applying selective criteria and propensity matching scores, this retrospective study demonstrated that viremic cases with HCC undergoing DAA therapy showed amelioration of liver function and a longer survival rate after viral eradication [5]. 

In conclusion, many aspects that drive the onset of HCC in cases with LTR after DAA administration remain unsolved, implying a more intimate cellular mechanism, for example, the unreversibility of the genetic tumor risk signature [15], the HCV synthesis or alteration of circulating miRNAs [16], or more intriguing modifications of the neo-angiogenetic transcriptomic signature [17] or the microenvironment and immune system [18]. 

Therefore, the hypothesis of sequential events favoring the onset of HCC after DAA administration may be rational, and some biomolecular events, even indirectly triggered by HCV or DAA administration in cases with an aptitude for more clinically advanced liver disease and/or biological genetic signatures, could be crucial for the acquisition of HCC status, even after a long-term response to DAA administration.

**The Main Take-Home Message from These Studies Are The Following**

(a) The benefit of DAAs in the treatment of CHC is closely related to the patient’s stage of liver fibrosis (Figure 1), with the highest incidence of HCC found especially in cases with clinically significant portal hypertension.

(b) DAAs do not eliminate HCC risk even in cases with LTR; thus, careful surveillance for HCC is mandatory and must offer better and more radical intervention for all cases, which often involves the patients’ listing for liver transplantation.

(c) Non-invasive scoring systems mainly reflecting hepatic function must be associated with standard fibrosis staging methods (i.e., measurement of liver and spleen stiffness by TE) for the identification of patients at higher risk of de novo HCC during long-term FU after DAA administration.

## Figures and Tables

**Figure 1:**
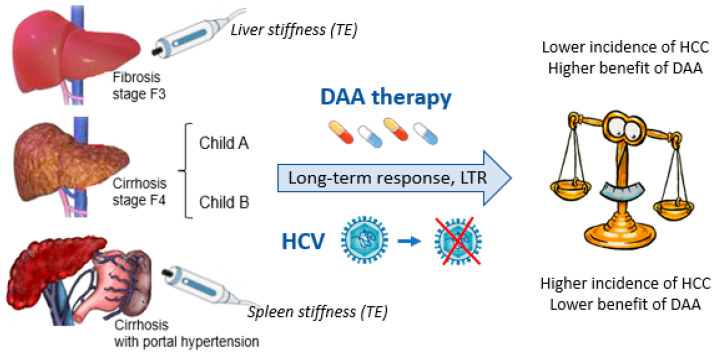
Benefits of DAA administration and HCC risk in cases with LTR: The close relationship with the patient’s stage of fibrosis.
